# Students’ educational needs for clinical reasoning in first clerkships

**DOI:** 10.1007/s40037-012-0010-7

**Published:** 2012-04-04

**Authors:** Thijs T. Wingelaar, Judith M. Wagter, Alf E. R. Arnold

**Affiliations:** Forest Medical School at the Medical Centre Alkmaar, PO Box 501, 1800 AM Alkmaar, the Netherlands

**Keywords:** Clerkships, Clinical reasoning, Educational needs, Qualitative research

## Abstract

Developing clinical reasoning skills early in medical education is important. However, research to uncover students’ educational needs for learning clinical reasoning during clerkships is limited. The aim of our study was to investigate these needs. Focus group discussions with an independent moderator were conducted. Students were included directly after 10 weeks of clerkships. The (verbatim) transcripts were coded manually and discussed by the authors until consensus was reached. Saturation was reached after three focus groups, including 18 students in total. Statistical analysis indicated our sample matched the approached group of 61 students. After a consistency and redundancy check in ATLAS.ti, 79 codes could be identified. These could be grouped into seven key themes: (1) transition to the clinical phase, (2) teaching methods, (3) learning climate, (4) students’ motivation, (5) teacher, (6) patient and (7) strategies in clinical reasoning. Students can adequately describe their needs; of the seven key themes relevant to clinical reasoning five are in line with literature. The remaining two (patient factors and the need for strategy for clinical reasoning) have not been identified before.

## Introduction

Clinical reasoning is a key competence of medical practice [1]. Clinical reasoning is a combination of analytical and non-analytical processes [2]. The analytical processes (hypothetico-deductive reasoning and scheme-inductive reasoning with flow charts and decision trees) play a major role early in the development of medical expertise, while the non-analytical processes (i.e. pattern recognition) play a more important role in clinical reasoning of experts [3, 4]. Scheme-inductive reasoning facilitates forward reasoning from symptoms to diagnosis and was shown to be associated with superior diagnostic success in both experts and novices than hypothetico-deductive reasoning [3]. There is evidence that non-analytical reasoning using pattern recognition develops over time when offered in an appropriate context [4].

Clinical experts regularly make use of compiled encapsulated knowledge (‘illness scripts’) in their diagnostic processes [1, 5]. Generated after previous patient encounters, illness scripts are committed to memory and are retrieved when physicians are faced with similar clinical problems in the future.

The compilation of knowledge and skills in illness scripts is thus a process that needs deliberate practice as was shown in other fields of expertise [6]. One can learn more, and more efficiently, from a structured way of studying cases or reflecting on contact with patients than without the structure or feedback [1, 7, 8].

At the start of the students’ clerkships (junior clerks), their needs for education in clinical reasoning are not necessarily congruent with those of undergraduates, interns or residents. Surprisingly, research to identify the educational needs of junior clerks for learning clinical reasoning is limited. Knowledge of educational needs could be relevant in order to construct effective educational programmes in clinical reasoning. The aim of our study is to explore junior clerks’ needs for learning diagnostic clinical reasoning. Focus groups were applied since they can be particularly useful to explore poorly understood or ill-defined topics [9, 10].

## Methods

### Study context

At the VU University Medical Center (Amsterdam, the Netherlands) clerkships start after 3 years of problem-based preclinical education. The following clinical phase (another 3-year period) leads to a medical degree and a basic qualification to practice medicine. The first year of clerkships is a combination of workplace learning and regular theoretical exams and is mainly designed to put communicative and manual skills into practice and to acquire clinical reasoning skills.

### Population

Directly upon completion of 10 weeks of clerkships (6 weeks internal medicine and 4 weeks surgery) in the period from Oct 2009 to Dec 2009 all students were invited to participate in this study. Participation was on a voluntary basis. This study was performed after 10 weeks of clerkships to ensure students have some experience in clerkships and are still able to reflect on this very first period of clerkships.

### Focus groups

An experienced and independent (i.e. not involved in the design of the study) moderator facilitated the semi-structured focus group discussions that each lasted ~120 min, while the main researcher took notes and asked questions to clarify points wherever necessary. This was concordant with general guidelines for conducting focus groups [11]. At the start of the session the moderator explained the procedure and guaranteed full confidentiality. To start the discussion, the moderator asked the junior clerks to write down some examples where they had learned clinical reasoning in the past 10 weeks. The definition of clinical reasoning was given in the introduction to make sure all participants used the same definition. Next, the participants were asked to explore the following key questions:

1. How did you experience these moments of learning?

*Introductory questions to familiarize students with the topic.*

2. What factors influenced your learning process (positive and negative)?

*To reflect on the previous question and to make a transition to the next question.*

3. How should ideal education in clinical reasoning be designed?

*This question reflects on the educational needs for learning clinical reasoning.*

Between question 2 and 3 the main researcher summarized the discussion and wrote key points down on a flip-over. The participants were asked if these points were correct and whether items were missing.

### Informed consent

The Medical Ethics Committee affiliated with the Medical Centre Alkmaar was consulted. They approved our methods for handling personal details and privacy and concluded they were concordant with the guidelines of the Association of Universities in the Netherlands. All participants received an informed consent form by e-mail a week before the focus group and signed a paper copy before the discussion was started.

### Analysis

All focus group recordings were transcribed verbatim and analyzed manually by all authors. This process commenced while data collection was in progress in order to tweak the topics of later focus groups and explore or check interesting findings. The transcripts of the first two focus groups were analyzed, assigning distinct codes to each remark made in relation to the research questions. During this process codes were renamed, reorganized and grouped into themes. Following transcripts were to be analyzed separately to check if the codes were consistent and no additional themes could be identified (saturation) [12]. To check for redundancy and consistency of our coding, the transcripts, remarks and codes were entered into qualitative data analysis software (ATLAS.ti) [13]. The remarks, codes and themes were cross-checked and discussed by the researchers until consensus was reached. The final step of the analysis included comparison of our themes with the literature.

## Results

We conducted three focus group discussions in the autumn of 2009, after which saturation was reached. Of the 61 students who met our inclusion criteria, 22 (36 %) responded of whom 4 eventually withdrew due to logistic difficulties. Only three students could attend the second focus group. A minimum of six participants is advised by most guidelines to ensure there is enough discussion. After analyzing the transcripts the data were similar to the other two focus groups (with 7 and 8 participants) and therefore included in the analysis. Of the 18 students who participated in our focus groups, 6 (33 %) followed their clerkships at a university medical centre and 12 (67 %) at a large teaching hospital. When we checked if our sample matched the approached group by comparing sex, age and type of hospital with a Pearson’s χ^2^ test, no statistical difference was found.

After a redundancy and consistency check in ALTAS.ti, 79 codes could be identified. Using the transcripts, these codes could be grouped into seven key themes relevant to learning clinical reasoning; (1) transition to the clinical phase, (2) teaching methods, (3) learning climate, (4) students’ motivation, (5) teacher, (6) patient and (7) strategies in clinical reasoning. The first three describe contextual factors (time, form and place) and the last one specifically refers to the contents of the reasoning process. The remaining three themes identify key figures in the process of learning clinical reasoning. Illustrating remarks on each key theme are presented in Table 1.

**Table 1:** Key themes (with the corresponding number of codes) with illustrative remarks (identified as Rx;Fy, where x refers to the respondent and y indicates the focus group)

Key theme (No. of codes)	Illustrative remarks
Transition to the clinical phase (4)	‘Textbooks aren’t always useful, because they start from diseases, not symptoms.’ R1;F1
	‘During the preclinical phase we followed the course on communication skills, but the focus was on the technique of conversation, not so much on the generation of differential diagnoses.’ R3;F2
Teaching methods (17)	‘You can’t exclusively learn from textbooks, you’ve got to witness it yourself. I think the combination is strong.’ R2;F1
	‘You just have to say something, because you’re in a group of 12 students. […] When the teacher asks a question he looks you in the eyes. So, I think that it has an effect on me.’ F2;R1
Learning climate (10)	‘It does make a difference. You are just one of the almost 40 clerks, interns and residents. As the most junior one you don’t have a lot of credit.’ R1;F3
Student (9)	‘And then you realize—if I forget to ask something now, it may be overlooked entirely. It feels much more my own responsibility.’ R1;F2
	‘[…] so I focus on the hassles and spend hours in the library rather than doing that what matters most: participating in the clinic’ R2;F2
	‘I come to drag up the story after the patient has been seen by so many doctors, residents and interns. So I finish off quickly in order to wrap up my presentation as soon as possible. I’m not going to bother this patient needlessly.’ R1;F1
Teacher (13)	‘To have a physician on your side who observes your history-taking or physical examination and puts you back on track when you stray off. Getting feedback afterwards is really different from getting direct feedback.’ R4;F3
	‘We barely see our teacher.’ R2;F3
	‘When we want to see her we have to go to the operating rooms. Then it’s clear that teaching junior clerks is not her priority and more an obligation.’ R3;F3
Patient (9)	‘Don’t you have patients who blurt out their assumed diagnosis without me having asked them a single question about it?’ R1;F3
	‘Yes.’ R7;F3
	‘That’s the major problem. […] You aren’t taking a history, you are listening to a patient’s story.’ R3;F3
	‘By telling patients in advance: ‘I’m a junior clerk, that means I’m in training, could you hold back your diagnosis so I can try to figure it out myself’. That works really well.’ R6;F3
Strategies in clinical reasoning (13)	‘I noticed my differential diagnosis came afterwards. I started connecting the dots: ‘these symptoms are linked with these diseases’. I think it’s hard to come up with possible diagnoses during my history-taking.’ R5; F3

Note that the number of codes in total is more than 79, because some codes relate to more than one theme

### Transition to the clinical phase

Junior clerks experienced the first clerkships as an abrupt transition from the preclinical to the clinical phase regarding clinical reasoning. Students thought the education in the preclinical phase was mainly focused on gathering knowledge. Signs and symptoms were explained through pathophysiological concepts. In the clinical setting the exact opposite was asked for; they had to learn to recognize signs and symptoms, and link these to possible diseases. A common opinion amongst the interviewed junior clerks was the preference for textbooks written from the viewpoint of signs and symptoms, not diseases.

### Teaching methods

Students recognized many different educational settings in which they learned clinical reasoning. Firstly, students mentioned involvement in daily practice. Assignments referring to patients they had seen, such as giving a case presentation for fellow students or making a status chart, helped them to build and structure their knowledge provided they got feedback on their performance. Secondly, the introductory course preceding the clinical phase (3 weeks of specific training in taking a history, physical examination and communication skills) was considered very useful as it provided the students with a set of tools to work with during clerkships. Lastly, the students considered lectures as a way to learn clinical reasoning only when it was an interactive session (preferably case presentations) in small groups.

### Learning climate

Students mentioned the difficulty of their position in daily practice. As the most novice participants in the workplace they sometimes felt intimidated and would refrain from asking questions. The interviewees noted a difference in culture between different departments and hospitals.

### Students’ motivation

The intake of new patients triggered students’ intrinsic motivation, because students saw their performance as decisive and felt more responsible for the patient’s health. They said that in those cases they felt there was no room for error and wanted to perform flawlessly. Conversely, the junior clerks mentioned they felt uncomfortable when doing redundant work (such as taking a history from a patient who has already been seen by specialists, residents and interns), because of the burden on the patient without clinical significance. Junior clerks reported that too much pre-knowledge on a case (i.e. knowing the diagnosis) reduced the motivation to take a thorough history and discouraged forward reasoning from symptom to diagnosis.

### Teacher

The ability to give adequate feedback on the process of clinical reasoning was mentioned as the main competence of a good teacher in clinical reasoning. A way to provide this was to observe the students while they were taking a history or physical examination and ask them to ‘think out loud’ or challenge them by asking questions. In this early phase of clinical reasoning students preferred a teacher who helped the student with a follow-up question when they appeared stuck in their history-taking. Students did not spontaneously mention a difference between residents and specialists in relation to learning clinical reasoning. Lack of time or enthusiasm on the part of the clinical teacher was considered a bigger influence, because this resulted in little or no contact between student and teacher and reduced the quantity or quality of feedback. Some hospitals had a dedicated teacher whose main task it was to guide and look after the well-being of the junior clerks. Junior clerks experienced this as a great advantage supporting their learning process, because the teacher could provide tailor-made arrangements if necessary or desirable (i.e. schedule attendance at interesting surgeries).

### Patient

Students preferred real patients over simulated (i.e. actor in a training setting) ones. Outpatients, if willing and able, could be asked to ‘simulate’ their first presentation with students taking a history and physical examination. Students reported that these patients often revealed their complete medical history after a few questions in order to help the student out. This was perceived as a threat to clinical reasoning. Students reported that a short instruction to the patient (either given by a teacher or themselves) not to reveal information spontaneously could prevent this.

### Strategies in clinical reasoning

All junior clerks perceived the simultaneous application of communication skills and cognitive aspects of clinical reasoning during history-taking as difficult. Most of the time students relied on lists of standard questions. Despite preparation by reading the referral letter or a short introduction by the clinical teacher the students hardly ever formed a differential diagnosis during the conversation with the patient. This was mostly done afterwards while reviewing the data by matching symptoms to known diseases (analytical reasoning). They shared the opinion that knowledge on pathophysiology was essential for generating a differential diagnosis. A few students mentioned their clinical reasoning needed more structure—a strategy to support them in clinical decision-making. No consensus could be reached regarding the contents of this strategy. Junior clerks that used a strategy for clinical reasoning reported their differential diagnosis consisted of more possible (accurate) diagnoses than without a strategy. Other students thought a uniform strategy could not suit their individual needs and would limit their reasoning capacities by forcing them to follow specific steps.

## Discussion

A considerable number of the approached students responded and participated in our study. We feel that this indicates that students are eager to contribute to the development of their education. It is apparent from this study that students are able to describe their educational needs for learning clinical reasoning. Their needs can be categorized into seven key themes: (1) transition to the clinical phase, (2) teaching methods, (3) learning climate, (4) students’ motivation, (5) teacher, (6) patient and (7) strategies in clinical reasoning. The last two are newly identified, the remaining five are in line with earlier reports in the literature. Inductive analysis on qualitative material gathered in focus groups proved to be a powerful method to explore needs [14–16].

### Transition to the clinical phase

Students participating in this study followed a curriculum with problem-based learning in small groups with integration of preclinical and clinical education. In addition, they were trained in clinical skills during a 3-week training in a skills laboratory. Nevertheless, students felt poorly prepared for patient encounters in the clinical setting. Students agree with the principles of deliberate practice when learning clinical reasoning [4, 17–19]. In concordance with the literature, students feel knowledge of basic science supports clinical reasoning [5, 20, 21].

### Teaching methods

Many different settings to develop clinical reasoning, i.e. simulation [17], case presentations [21] and demonstration [22], have been recognized by our respondents. Consistent with previous research our students mention that these moments require interaction between teacher and student, proper feedback and an authentic setting [17, 21–23].

### Learning climate

The main aim of medical curricula is to provide students with the necessary materials and experiences to facilitate individual learning. The findings of this study concerning the learning climate (culture and the possibility to participate in daily practice) are consistent with many studies [24, 25].

### Students’ motivation

The contributors to the focus groups indicated the importance of factors that improve the intrinsic motivation for learning. Intrinsic motivation is known to be one of the key factors of deep learning. It is known that learners who use deep approaches in learning tend to enjoy the experience, retain more factual material over a longer period and demonstrate higher quality outcomes and better grades [26]. Junior clerks mentioned pre-knowledge on a diagnosis decreased their drive to take a history or physical examination, as described in literature [27].

### Teacher

The characteristics we identified for a good clinical teacher (constructive feedback based on observations, suggesting a follow-up question when students are stuck in their history-taking and asking students to ‘think out loud’) are in line with the literature [23, 28, 29].

### Patient

If diagnostic reasoning tasks are performed on (out) patients who already know their diagnosis, instruction not to reveal information on the diagnosis promotes forward reasoning. We feel this type of patient contact, with the proper instruction, approaches the authentic clinical setting and could be used to teach clinical reasoning in junior clerks.

### Strategies in clinical reasoning

Although pattern recognition results in higher diagnostic accuracy, this approach is not an appropriate learning tool for students. Scheme-inductive reasoning may be the optimal way to teach and learn diagnostic reasoning since schemes (i.e. flow charts and decision trees) reflect an organized knowledge structure for learning and teaching diagnostic reasoning [30]. Prefabricated schemes are not always useful, as self-made schemes generate the clinically relevant chunks of information in memory most efficiently.

## Implications

### Multi-tasking

Since students struggle with the simultaneous execution of communicating with a patient and the cognitive aspects of clinical reasoning, it seems reasonable that education to combine these two skills should be introduced early in the curriculum [31]. The delayed generation of a diagnosis (until after the conversation) stresses that patients with a life-threatening condition should never be seen by clerks without hands-on supervision.

### Patient factors

When junior clerks are practising clinical reasoning skills (out) patients who already know their diagnosis can be asked to ‘simulate’ their first presentation. Although this may seem an authentic learning environment, this setting does not promote intrinsic motivation similar to the intake of a new patient.

### Strategies in clinical reasoning

Junior clerks seem to prefer an analytical approach to clinical reasoning, but the offered strategy must leave enough room for individual variation. The students’ preference for textbooks written from a ‘symptoms’ perspective identified in this study could help course designers when selecting an apt textbook for their junior clerks.

## Strengths and limitations

The qualitative method used in this study helps us to understand the obstacles encountered by junior clerks and can contribute to the development of ‘tailor-made education’. Because we reached saturation and our sample statistically matched the approached group, the results of this study are applicable to our population. Although we are inclined to expect similarities in medical students starting their clerkships at other universities or in other countries, the generalizability of our findings should be assessed in future research.

## Conclusion

This study underscores the importance of learning in the clinical setting to enhance the intrinsic motivation by taking care of real patients. The clinical workplace is the forum for forward reasoning and sets the stage for learning to combine history taking and clinical reasoning simultaneously.

## Essentials

- Junior clerks struggle with the *simultaneous* execution of communication and reasoning skills.
- ‘Patient factors’ should be taken into account when junior clerks are practising clinical reasoning.

## References

[1] Norman G (2005). Research in clinical reasoning: past history and current trends. Med Educ.

[2] Eva KW (2005). What every teacher needs to know about clinical reasoning. Med Educ.

[3] Coderre S, Mandin H, Harasym PH, Fick GH (2003). Diagnostic reasoning strategies and diagnostic success. Med Educ.

[4] Norman G, Young M, Brooks L (2007). Non-analytical models of clinical reasoning: the role of experience. Med Educ.

[5] Schmidt HG, Norman GR, Boshuizen HP (1990). A cognitive perspective on medical expertise: theory and implication. Acad Med.

[6] Ericsson KA, Charness N (1994). Expert performance: its structure and acquisition. Am Psychol.

[7] Bowen JL (2006). Educational strategies to promote clinical diagnostic reasoning. N Engl J Med.

[8] Schuwirth L (2002). Can clinical reasoning be taught or can it only be learned?. Med Educ.

[9] Barbour R (2005). Making sense of focus groups. Med Educ.

[10] Kitzinger J (1995). Qualitative research. Introducing focus groups. BMJ.

[11] Morgan DL (1997). Focus groups as qualitative research.

[12] Robson C (2002). Real world research.

[13] ATLAS.ti [computer program]. Version 5.2. Berlin: Scientific Software Development; 1999.

[14] Pope C, Ziebland S, Mays N (2000). Qualitative research in health care: analysing qualitative data. BMJ.

[15] Dingwall R, Murphy E, Watson P, Greatbatch DSP, Parker S (1998). Catching goldfish: quality in qualitative research. J Health Serv Res Policy.

[16] Murphy E, Dingwall R, Greatbatch DSP, Watson P. Qualitative research methods in health technology assessment: a review of the literature. Health Technol Assess. 1998;2(16):iii–ix, 1–274. 9919458

[17] Kneebone RL, Scott W, Darzi A, Horrocks M (2004). Simulation and clinical practice: strengthening the relationship. Med Educ.

[18] Bloch RF, Hofer D, Feller S, Hodel M (2003). The role of strategy and redundancy in diagnostic reasoning. BMC Med Educ.

[19] Rikers RMJP, Loyens S, Te WW, Schmidt HG, Sins PHM (2005). The role of biomedical knowledge in clinical reasoning: a lexical decision study. Acad Med.

[20] Woods NN, Brooks LR, Norman GR (2005). The value of basic science in clinical diagnosis: creating coherence among signs and symptoms. Med Educ.

[21] Onishi H (2008). The role of case presentation for teaching and learning activities. Kaohsiung J Med Sci.

[22] Borleffs JCC, Custers EJFM, Van Gijn J, Cate OT (2003). ‘Clinical reasoning theater’: a new approach to clinical reasoning education. Acad Med.

[23] Steinert Y (2004). Student perceptions of effective small group teaching. Med Educ.

[24] Teunissen PW, Scheele F, Scherpbier A (2007). Residents’ perspectives on their learning. Med Educ.

[25] Boor K, Teunissen PW, Scherpbier A, Vleuten C, van der Lande J, Scheele F (2008). Residents’ perceptions of the ideal clinical teacher—a qualitative study. Eur J Obstet Gynecol Reprod Biol.

[26] Busari JO, Arnold AER (2009). Educating doctors in the clinical workplace: unraveling the process of teaching and learning in the medical resident as teacher. J Postgrad Med.

[27] Dhaliwal G, Sharpe B (2009). Twelve tips for presenting a clinical problem solving exercise. Med Teach.

[28] Alweshahi Y, Harley D, Cook DA (2007). Students’ perception of the characteristics of effective bedside teachers. Med Teach.

[29] Paukert JL, Richards BF (2000). How medical students and residents describe the role and characteristics of their influential clinical teachers. Acad Med.

[30] Mandin H, Jones A, Woloschuk W, Harasym P (1997). Helping students learn to think like experts when solving clinical problems. Acad Med.

[31] Windish DM, Price EG, Clever SL, Magaziner JL, Thomas PA (2005). Teaching medical students the important connection between communication and clinical reasoning. J Gen Intern Med.

